# Supplementary material for: Learning curve and surgical outcome of robotic assisted colorectal surgery with ERAS program
Source: Sci Rep. 2022 Nov 29;12:20566. doi: 10.1038/s41598-022-24665-w (PMC9709162; doi:10.1038/s41598-022-24665-w)
Supplement: Supplementary file 2 — Supplementary Information 2. [file 41598_2022_24665_MOESM2_ESM.zip › RAL Raw data-Ñ[▒K/101/HS_ñw╝╨░O▒Kñσ.pdf]

正式摘要(電子簽章：000000224351500)
W52 / W52 ☐英文標題(注意:無英文姓名-請補病人基本檔!) ☐【讀取】憑證

臺中榮民總醫院

出院病歷摘要

轉入號： 轉出號：

[redacted]

(9)入院日期	110/07/27 (2021) 直腸外科 W52 - 101
(10)轉科日期	
(11)出院日期	110/09/06 (2021) 住院天數計 41 日

診斷

- 入院 :1. Rectal Cancer,4cm FAV c T3 N1 M0, Stage IIIB
  CCRT (#VB1 56Gy/28frs) since 2021/05/10
  with FOLFOX on 2021/5/8, 5/23
  with 5-FU on 2021/6/12, 7/04
  2. peri-anal abscess, s/p Fistulectomy 2020/10/30
  3. HCV carrier
- 出院 :1. Rectal Cancer,4cm FAV c T3 N1 M0, Stage IIIB
  -CCRT (#VB1 56Gy/28frs) since 2021/05/10
  -with FOLFOX on 2021/5/8, 5/23
  -with 5-FU on 2021/6/12, 7/04
  -s/p Robotic (Xi) assisted TME + loop ileostomy on 2021/07/28
  -with IAI, s/p Brosym 2021/08/06-08/27, Fluconazole 2021/08/15-08/30, Colistin 2021/08/27-09/01, Tigecycline 2021/09/01-09/06 ,
  s/p PCD insertion on 2021/08/18, removed on 2021/09/04
  ypT3N1c(0/18)M0, MD, AI-, PI-, TuDp+3, CRM 7mm -, ExNd-, TRG: no 3
  stage IIIB
  2. peri-anal abscess, s/p Fistulectomy 2020/10/30
  3. HCV carrier

主訴

- for scheduled surgery

病史

- the 50 year-old male had history of
  1. Rectal Cancer,4cm FAV c T3 N1 M0, Stage IIIB
  CCRT (#VB1 56Gy/28frs) since 2021/05/10
  with FOLFOX on 2021/5/8, 5/23
  with 5-FU on 2021/6/12, 7/04
  2. peri-anal abscess, s/p Fistulectomy 2020/10/30
  3. HCV carrier

  He suffered from poor appetite, body weight loss(10kg in a year) for one year and went to our CRS OPD. DRE showed palpable mass over posterior wall. 2021/4/19 CT revealed rectum tumor. 4/21 colonscopy showed rectum tumor 4cm FAV, s/p biopsy. Pathology later yielded Adenocarcinoma, moderately differentiated. 4/29 Pelvis MRI: wall thickening appearance over rectum, about 5.2 cm in length, rectal cancer could be compatible. PET on 5/7 showed increased FDG uptake over rectal region, pararectal region, left upper abdomen, gastric region, thoracic esophagu. 1st C/T with FOLFOX was done on 2021/5/8. To r/o gastric metastasis, we arranged UGI endoscopy, s/p biopsy over stomach, esophagus which pathology had no malignant findings. We also check HCV RNA viral load for HCV carrier status evaluation, which was unremarkable. The patient has began R/T since 2021/5/11. He complained of anal skin pain under current R/T.
  He finished chemotherapy course as mentioned above. He was admitted this time for scheduled therapy.

體檢發現

-
  - General appearance :
    - easy looking , conscious clear , without cardiopulmonary distress
    - Vital signs自動帶入date 體溫(BT) 脈搏(P) 呼吸(R) 血壓(BP) 身高(BH) 體重(BW) BMI
      20210727 36.1℃ 81次/分 18次/分 109/87mmHg 169.4cm 73.4kg 25.6
    - Vital signs: B.P. 129/78 mmHg , P.R. 76 /min , R.R. 16 /min , B.T. 36.1 ℃
  - HEENT :
    - Eyes: no anemic conjunctiva, no icteric sclera, normal EOM
    - ENT: no otorrhea, no rhinorrhea, no erythematous mucosa, no injected tonsil, no deformity
  - Neck: supple, freely movable, trachea midline,no deformity, no engorged jugular vein , no palpable thyroid or lymph node enlargement
  - Chest:
    - Inspection: Grossly normal, symmetric and fully expansion, no accessory muscle used, no operation scar, no spider angiomata, no gynecomastia
    - Palpation: no palpable mass or nodule, no subcutaneous emphysema, no tenderness
    - Ausculation: clear breathing sound

-
  - Heart:
    - Palpation: no thrill, no heave
    - Percussion: normal heart size
    - Ausculation: regular heart beat, normal S1, S2, no gallop, no murmur
  - Extremities: No deformity, full ranged of motion without limitation, no clubbing finger or nail cyanosis, no pitting edema, no ulcer or pigmentation, no obvious skin lesion, no varicose vein, no bone, joint or muscle tenderness

CRS CONDITION

- Karnofsky performance status: (100) Normal, no complaints, no evidence of disease
- body weight : 73 kg , height : 168 cm
- CEA: 46.42 ng/ml

手術日期及方法

- 手術日期及方法20210728 1. Robotic (Xi) assisted TME + loop ileostomy
  2. Intraoperative colonoscopy : (-).
  3. High ligation (+) : At IMA root. IMV was also ligated.
  4. Take down from the splenic flexure : (-)
  5. Robotic EndoGIA (45-4.3) X4 + EEA (29mm) were applied to complete the end-to-end anastomosis
  6. Proximal cut end: > 10 cm, distal cut end: 0.5 cm
  7. A 12 Fr. penrose drainage tube was inserted from RLQ of abdomen (8mm trochar site) into anastomosis (Pelvic) area
  8. Loop ileostomy (About 15~20cm from IC valve) was done over LLQ of abdomen

住院治療經過

- After admission, the patient received robotic (Xi) assisted TME + loop ileostomy on 2021/07/28. After operation, abdominal pain and vomitus was found. KUB disclosed ileus, improved after antibiotics, NPO and NG decompresison treatment, and he started to try diet after symptoms subsided. Fever episode on 8/7 was noted, fever survey disclosed ascites bacteria, s/p anbitiotics treatment. However, intermittent fever episodes happened after we changed antibiotics. To r/o intra-abdominal infection, we arranged abdominal CT on 8/17, which disclosed intraabdominal abscess at presacral and left paracolic gutter. PCD insertion was done on 8/18. We've also adjsuted antibiotics according to his ascites culture report, with Brosym 2021/08/06-08/27, Fluconazole 2021/08/15-08/30, Colistin 2021/08/27-09/01, Tigecycline 2021/09/01-09/06. Fever subsided after PCD drainage and antibiotics. Due to decreased drainage amount, we pulled out PCD for 6cm on 9/3 and removed it on 9/4. Besides, he had urinary retention with recurrent foley's tube insertion, suspect acute urinary retension after post-pelvic surgery and pain related, s/p alpha blockers. Foley's tube was removed smoothly on 9/3 with smooth self-urination. He also had adequate nutrition and defecation condition. Under stable condition, he was discharged today. OPD follow up was arranged.

合併症

一般檢查

- 累積報告 :

DATE	ALB	TP	BIL.T	BII.D	ALKP	AST	ALT	LDH	CHOL	TG	HDL-C	LDL	UA	NH3	AMY
1011107			2.1			190	81								
1020204			0.7			46	67								
1020520	4.6		1.1			73	82								
1020826			0.7			98	117								
1030508			1.9			41	36								
1030811			0.5			39	85								
1031103			1.1			104	85								
1040105			0.9			44	90								
1040413			0.5			72	172								
1040522			0.7			31	70								
1040810			0.5			35	61								
1041102						28	50								
1050125			0.6			26	35								
1050614			0.8			27	42								
1050907			0.7			29	59								
1051125			1.0			30	42								
1060301			1.1			28	55								
1091030			1.3				43								
1100419						71	43								
1100517						16	16								
1100611	4.0	6.3	0.35	0.13	59	11	11	164							
1100703			0.3			14	12	185							
1100721	4.4		0.70	0.18	45	16	12	180							
1100728	3.6		0.8			16	8								
1100729			1.1				6								
1100802	2.5		2.19	1.20		16	15								
1100805	2.8		1.51	0.76		14	7								
1100808	3.5		1.33				10								
1100812	2.8		1.29	0.59											
1100816	3.1		0.92	0.44		165	110								
1100826	3.3		0.63				74								
1100902	3.5		0.72	0.29		41	66								
1100906			0.77	0.30		20	37								

DATE	ALB	TP	BIL.T	BII.D	ALKP	AST	ALT	LDH	CHOL	TG	HDL-C	LDL	UA	NH3	AMY
1011107			2.1			190	81								
1020204			0.7			46	67								

1020520	4.6		1.1			73	82								
1020826			0.7			98	117								
1030508			1.9			41	36								
1030811			0.5			39	85								
1031103			1.1			104	85								
1040105			0.9			44	90								
1040413			0.5			72	172								
1040522			0.7			31	70								
1040810			0.5			35	61								
1041102						28	50								
1050125			0.6			26	35								
1050614			0.8			27	42								
1050907			0.7			29	59								
1051125			1.0			30	42								
1060301			1.1			28	55								
1091030			1.3				43								
1100419						71	43								
1100517						16	16								
1100611	4.0	6.3	0.35	0.13	59	11	11	164							
1100703			0.3			14	12	185							
1100721	4.4		0.70	0.18	45	16	12	180							
1100728	3.6		0.8			16	8								
1100729			1.1				6								
1100802	2.5		2.19	1.20		16	15								
1100805	2.8		1.51	0.76		14	7								
1100808	3.5		1.33				10								
1100812	2.8		1.29	0.59											
1100816	3.1		0.92	0.44		165	110								
1100826	3.3		0.63				74								
1100902	3.5		0.72	0.29		41	66								
1100906			0.77	0.30		20	37								

特殊檢查

病理報告

- 活體組織切片病理報告 手術日期：**110/07/28** 病理號：**S1023072**

診斷醫師:李欣倪 報告日期:110/08/03
Pathologic diagnosis:
1. Rectum, robotic low anterior resection --- Adenocarcinoma, moderately differentiated. Tumor invades to perirectal soft tissue with tumor deposits. The bilateral cut ends and perirectal lymph nodes (0/16) are free of tumor.
2. Rectum, cut end, distal, resection --- Chronic inflammation.
3. IMA root, dissection --- Unremarkable fibrovascular tissue and lymph nodes (0/2).

Ancillary study for diagnosis:
IHC stains for PMS2 and MSH6 are done on section E. Both markers are preserved.
Prognostic and predictive factor:
1. Tumor size: 2.8x2 cm.
2. Depth of invasion: Tumor invades to pericolorectal tissue.
3. Histologic grade: G2: Moderately differentiated.
4. Angiolymphatic invasion: Not identified.
5. Perineural invasion: Not identified.
6. Tumor deposits (discontinuous extramural extension): Present, specify number of deposits: 3.
7. Macroscopic tumor perforation: Not identified.
8. Surgical Margins: a) Proximal Margin: Uninvolved by invasive carcinoma, distance of tumor from margin: 12.5 cm. b) Distal Margin: Uninvolved by invasive carcinoma, distance of tumor from margin: 1.8 cm. c) Radial or Mesenteric Margin: Uninvolved by invasive carcinoma, distance of tumor from margin: 7 mm.
9. Lymph node status: Uninvolved (0/18).
10. Extranodal involvement: Not identified.
11. Tumor regression grading S/P CCRT: No known presurgical therapy.
12. Type of polyp in which invasive carcinoma arose: Not identified.
13. Additional pathologic findings: Not identified.
14. Pathological TNM Stage: pT3N1c(According to the eighth edition, American Joint Committee on Cancer Staging Guidelines for Tumors).
15. TNM descriptors: Not applicable.

Gross description:
The specimen consists of 1) a segment of colon, 16.5 cm in length and 4 cm in proximal cut end circumference, 5.5 cm in distal circumference, and perirectal soft tissue. A gray tan firm annular elevated and centrally ulcerative tumor, 2.8x2 cm, is noted, 1.8 cm to the nearest cut end. On cross section, the tumor invades to the mesocolic soft tissue with 7 mm to the radial margin, and serosal retraction is also noted. The both cut ends are free of tumor

- grossly. 2) distal cut end, a piece of tan mucosa-covered soft tissue, 2.3x2.1x0.8 cm. 3) IMA root, a piece of tan yellow soft tissue, 2.4x1.1x0.9 cm. Representative part for section: A) proximal cut end B) distal cut end C-E) tumor F) colon G-H) specimen 2-3 J-L) lymph nodes.
  Note:
  1. The interpretation of MMR panel and recommended studies are as below: (1) No loss of nuclear expression of MMR proteins: low probability of microsatellite instability-high (MSI-H) . (2) Loss of nuclear expression of MLH1 and PMS2: testing for methylation of the MLH1 promoter and/or mutation of BRAF is indicated (the presence of a BRAF V600E mutation and/or MLH1 methylation suggests that the tumor is sporadic and germline evaluation is probably not indicated; absence of both MLH1 methylation and of BRAF V600E mutation suggests the possibility of Lynch syndrome, and sequencing and/or large deletion/duplication testing of germline MLH1 may be indicated). (3) Loss of nuclear expression of MSH2 and MSH6: high probability of Lynch syndrome (sequencing and/or large deletion/duplication testing of germline MSH2 may be indicated, and, if negative, sequencing and/or large deletion/duplication testing of germline MSH6 may be indicated). (4) Loss of nuclear expression of MSH6 only: high probability of Lynch syndrome (sequencing and/or large deletion/duplication testing of germline MSH6 may be indicated). (5) Loss of nuclear expression of PMS2 only: high probability of Lynch syndrome (sequencing and/or large deletion/duplication testing of germline PMS2 may be indicated). (6) Please note: There are exceptions to the above IHC interpretations. These results should not be considered in isolation, and clinical correlation with genetic counseling is recommended to assess the need for germline testing.
  2. This case has been peer reviewed by two doctors.
  Erratum: 2021-08-19
  Prognostic and predictive factor:
  11. Tumor regression grading S/P CCRT: Absent, extensive residual cancer with no evident tumor regression (poor or no response, score 3).
  14. Pathological TNM Stage: ypT3N1c(According to the eighth edition, American Joint Committee on Cancer Staging Guidelines for Tumors).
  15. TNM descriptors: y (post-treatment)
  #T-59300_2 #M-81403_2 2 1105 S0ER0F
  #T-C4560_2 #M-09183_2 0 1105 000000

其它

- 隔離註記:接觸防護【VRE(檢)(20210831),CRE(檢)(20210826)】

出院時情況

- 3.改門診治療

出院指示

藥名	劑量	劑量單位	用法	頻次	數量	數量單位	現況	天數	單位
Dobecon tab 100mg	1	TAB	PO	TIDAC	21	TAB	TH	7	天
Loperamide cap 2mg	1	CAP	PO	TID	21	CAP	TH	7	天
Voren-K tab 25mg	1	TAB	PO	TID	21	TAB	TH	7	天
CR Stilnox tab6.25	1	TAB	PO	HS	7	TAB	TH	7	天
BethanechoL tab 25mg-JS	1	TAB	PO	TIDAC	21	TAB	TH	7	天
TramTOR cap 50mg	1	CAP	PO	TID	21	CAP	TH	7	天
OCAS Harnalidge 0.4 mg	1	TAB	PO	QDAC	7	TAB	TH	7	天

出院計劃

- 門診日期
  - date on 2021/09/13

主治醫師_2222H 林俊余 / 住院醫師_9739F 趙雅芸

正式摘要(電子簽章：**000000224351500)**

W52 / W52

doctorRank:B
